# In vitro prediction of the efficacy of molecularly targeted cancer therapy by Raman spectral imaging

**DOI:** 10.1007/s00216-015-8875-z

**Published:** 2015-07-14

**Authors:** Hesham K. Yosef, Laven Mavarani, Abdelouahid Maghnouj, Stephan Hahn, Samir F. El-Mashtoly, Klaus Gerwert

**Affiliations:** Department of Biophysics, Ruhr University Bochum, Universitätsstr. 150, 44780 Bochum, Germany; Department of Molecular GI-Oncology, Clinical Research Center, Ruhr University Bochum, Universitätsstr. 150, 44780 Bochum, Germany

**Keywords:** Raman imaging, Erlotinib, Oncogenic mutation, *BRAF*, *KRAS*, Colon cancer

## Abstract

Mutational acquired resistance is a major challenge in cancer therapy. Somatic tumours harbouring some oncogenic mutations are characterised by a high mortality rate. Surprisingly, preclinical evaluation methods do not show clearly resistance of mutated cancers to some drugs. Here, we implemented Raman spectral imaging to investigate the oncogenic mutation resistance to epidermal growth factor receptor targeting therapy. Colon cancer cells with and without oncogenic mutations such as *KRAS* and *BRAF* mutations were treated with erlotinib, an inhibitor of epidermal growth factor receptor, in order to detect the impact of these mutations on Raman spectra of the cells. Clinical studies suggested that oncogenic *KRAS* and *BRAF* mutations inhibit the response to erlotinib therapy in patients, but this effect is not observed in vitro. The Raman results indicate that erlotinib induces large spectral changes in SW-48 cells that harbour wild-type *KRAS* and *BRAF*. These spectral changes can be used as a marker of response to therapy. HT-29 cells (*BRAF* mutated) and SW-480 cells (*KRAS* mutated) display a smaller and no significant response, respectively. However, the erlotinib effect on these cells is not observed when phosphorylation of extracellular-signal-regulated kinase and AKT is monitored by Western blot, where this phosphorylation is the conventional in vitro test. Lipid droplets show a large response to erlotinib only in the case of cells harbouring wild-type *KRAS* and *BRAF*, as indicated by Raman difference spectra. This study shows the great potential of Raman spectral imaging as an in vitro tool for detecting mutational drug resistance.

**Electronic supplementary material:**

The online version of this article (doi:10.1007/s00216-015-8875-z) contains supplementary material, which is available to authorized users.

## Introduction

Cancer is one of the leading causes of death worldwide. Statistics from the World Health Organization indicate that 8.2 million cases of cancer death were registered in 2012 [1]. This high number of cancer deaths is due to the failure of many therapeutic strategies to suppress tumour progression. Some oncogenic mutations render tumours aggressive, which is accompanied by poor prognosis and a decreased response to therapy [2–4]. Therefore, researchers in drug development are competing to find new potent anticancer drugs that can overcome the mutational drug resistance. Hundreds of new anticancer drugs appear each year. To evaluate their potencies, they are studied in a series of in vitro and in vivo preclinical tests. The promising candidates are then proceeded to clinical trials, to be tested on human cancer patients [5]. However, preclinical methods do not predict the resistance of some oncogenic mutations to therapy, which is later detected in many clinical studies. For instance, inhibitors of epidermal growth factor receptor (EGFR) showed great therapeutic potential in solid tumour suppression [6]. However, some of them failed in patients with *KRAS*-mutated cancers [7].

**Fig. 1:**
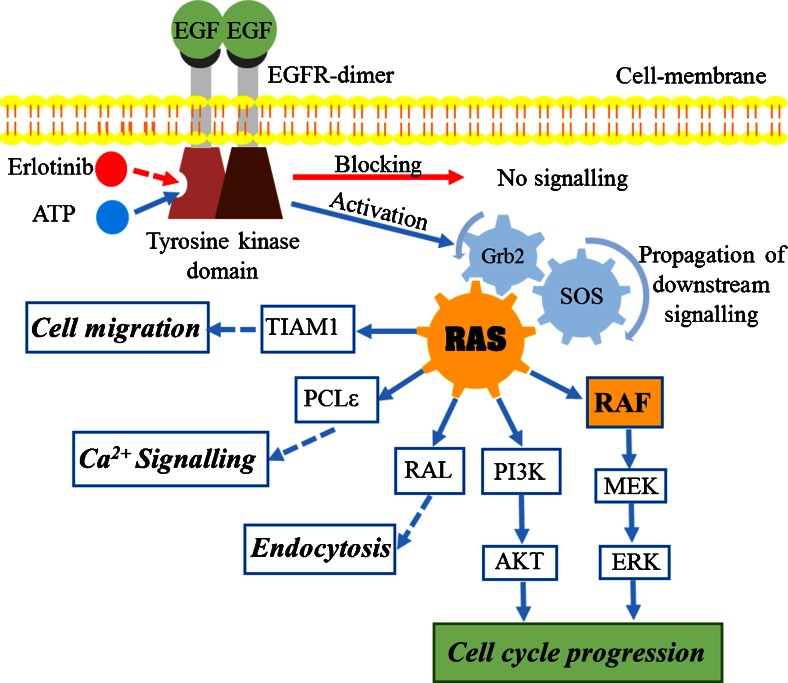
Epidermal growth factor receptor (*EGFR*)–RAS downstream signalling cascade

Owing to the overexpression of EGFR in many cancers, targeting EGFR is one of the effective strategies to suppress tumours in advanced stages [6,8]. EGFR is a transmembrane protein with extracellular, transcellular, and intracellular domains. It is a member of the ERBB family of receptors. Upon binding of a ligand such as epidermal growth factor (EGF), EGFR dimerises with other homologous members of the ERBB receptors, which promotes phosphorylation of the intracellular tyrosine kinase (TK) domain, which in turn stimulates cell cycle progression as shown in Fig. 1. Erlotinib (Tarceva) is a small molecule that inhibits the phosphorylation of the EGFR TK domain. It blocks downstream signalling transduction and tumour progression [9]. It is clinically approved by the US Food and Drug Administration and the European Medicines Agency for the treatment of advanced non-small-cell lung, and pancreatic cancers [10–15]. Erlotinib is still in clinical trials for the treatment of colon cancer [16].

RAS is a membrane-anchored guanosine triphosphatase protein [17]. It controls multiple downstream signalling pathways and acts as an on/off signalling switch (Fig. 1). Activated EGFR sets RAS to on mode, which in turn activates multiple effector proteins such as rapidly accelerated fibrosarcoma (RAF), phosphatidylinositiol 3-kinase (PI3K), TIAM, phospholipase Cε (PCLε), and RAL for cell proliferation and survival [17–19]. In the case of EGFR inhibition, RAS remains in off mode, and the cell cycle is arrested [20]. However, mutated RAS is chronically switched to on mode, and it becomes independent of EGFR activation. Therefore, RAS-mutated tumours are aggressive, invasive, and metastatic. Moreover, it is accompanied by a decreased response to EGFR-inhibition therapy [4,7,17,18,21–25]. *KRAS* mutation accounts for 86 % of all RAS mutations [26]. It is commonly found in the most deadly cancer types: lung (33 %), colon (40 %), and pancreatic (90 %) cancers [27,28]. *KRAS* mutation also is reported to be predictive for poor prognosis and low survival rate in cancer [22,25,29].

RAF is one of the well-identified RAS effector proteins, with serine/threonine kinase activity [17]. RAF is activated by binding with the active form of RAS, which subsequently stimulates the mitogen-activated protein kinase–extracellular-signal-regulated kinase (ERK) pathway proteins through a cascade of autophosphorylation events towards cell proliferation (Fig. 1) [30]. *BRAF* mutation occurred in up to 80 % of skin cancers and 5-10 % of colon cancers [31]. In addition, *BRAF* mutation is accompanied by an elevated kinase activity, which increases ERK phosphorylation [32,33]. Clinical studies showed that cancer patients with *BRAF* mutation have a relatively poor prognosis [25,34].

Oncogenic mutations are commonly detected by DNA sequencing and methods based on polymerase chain reaction [35,36]. In vitro assessment of drug effects is done separately by cytotoxicity assays [5]. Although, some in vivo methods such as genetically engineered cancer models revealed promising results in detecting drug resistance to mutations [37,38], they still have some drawbacks, such as requiring a long time and unpredictability of tumour formation [39]. To the best of our knowledge, no in vitro method has reported so far the impact of oncogenic mutations on response to EGFR molecularly targeted therapy.

Raman micro-spectroscopic imaging is an emerging technique in biomedical research. Raman spectroscopy can measure biological samples in an aqueous physiological environment. It is a label-free, non-invasive technique with high spectral/lateral resolution and great reproducibility [40–43]. Raman spectral imaging can classify cancerous human tissues [44,45]. It can be used for imaging of single cells and characterisation of subcellular components [46–50]. Furthermore, Raman imaging can be conducted to monitor drug uptake and its effect on single cells [51–57]. In our previous work we investigated the distribution and metabolism of erlotinib in SW-480 colon cancer cells using its unique –C ≡ C– band at 2100 cm^−1^, which is used as a marker band for erlotinib localisation [53].

Here, we implemented Raman imaging coupled with hierarchical cluster analysis (HCA) to monitor the response of colon cancer cells to erlotinib therapy. We report in vitro evidence that detects the effect of oncogenic *KRAS* and *BRAF* mutations on the cellular response to erlotinib. The Raman results show that colon cancer cells experience a large spectral response to erlotinib, but colon cancer cells expressing oncogenic *BRAF* or *KRAS* mutations experience small or no relevant effects, respectively. Furthermore, the largest effect is observed in lipid droplets of cancer cells harbouring wild-type *BRAF* and *KRAS* that were treated with erlotinib.

## Material and methods

### Cell culture

The colon cancer cell lines SW-48, HT-29, and SW-480 were purchased from American Type Culture Collection. Cells were cultured in Dulbecco’s modified Eagle’s medium (Life Technologies, Darmstadt, Germany) supplemented with 10 % fetal bovine serum (Life Technologies, Darmstadt, Germany), 2 mM l-glutamine, and 5 % penicillin–streptomycin, and were incubated at 37 °C in a 10 % CO_2_ atmosphere. Cells were subcultured to 80 % confluence, detached by trypsin–EDTA (0.25 %) (Gibco trypsin solution, Life Technologies, Darmstadt, Germany), centrifuged at 1500 rpm for 3 min and diluted to 10 %, then seeded again in culture medium. Raman measurements were performed on cells grown on CaF_2_ windows (Korth Kristalle, Kiel, Germany) to avoid Raman scattering from regular glass slides. Cells were incubated with erlotinib (Tarceva; Roche, Switzerland) at 10 μg/ml at 37 °C in 10 % CO_2_ for 12 h. Subsequently, cells were fixed in 4 % paraformaldehyde (VWR International, Darmstadt, Germany) and then submerged in phosphate-buffered saline (Life Technologies, Darmstadt, Germany).

### Confocal Raman microscopy

Cancer cells were measured with an alpha300 AR confocal Raman microscope (WITec, Ulm, Germany) as described previously [45,50,53]. Briefly, the setup excitation source was a frequency-doubled Nd:YAG laser of 532 nm (CrystaLaser, Reno, NV, USA) with an output power of around 40 mW. The laser radiation was coupled into a Zeiss microscope through a wavelength-specific single-mode optical fibre. An achromatic lens used as a collimator for the laser beam, and a holographic band-pass filter focused the beam on the sample through a Nikon NIR APO (×60/1.00 numerical aperture) water immersion objective. The sample was located on a piezoelectrically driven microscope scanning stage, which has an *x*,*y* resolution of 3 nm and a *z* resolution of 0.3 nm. Raman back-scattered light was collected through a microscopic objective and passed through a holographic edge filter into a multimode fibre (50-μm diameter) and into a 300-mm focal length monochromator, which incorporated a 600/mm grating blazed at 500 nm. A back-illuminated deep-depletion charge-coupled device camera operating at −60 °C was used to detect Raman spectra. We acquired Raman imaging measurements by raster scanning the laser beam over cells and collecting a full Raman spectrum at speed of 0.5 s per pixel, with a pixel resolution of 500 nm.

### Multivariate analysis

HCA and principal component analysis (PCA) were performed on Raman spectra of cells. Raman hyperspectral results were exported to MATLAB 8.2 (The MathWorks, Natick, MA, USA). Scripts written in-house were used to perform data preprocessing and multivariate analyses. Raman spectra without a C–H band at 2850–3000 cm^−1^ were treated as the background and were deleted. An impulse noise filter was used to remove cosmic spikes, and the Raman spectra were interpolated to a reference wavenumber scale. All spectra were then baseline corrected with a third-order polynomial and were also vector normalised. HCA was performed on the regions from 700 to 1800 cm^−1^ and 2800 to 3050 cm^−1^ with use of Ward’s clustering in combination with the Pearson correlation distance. All Raman mean spectra obtained from HCA were normalised with the phenylalanine band near 1008 cm^−1^. We calculated Raman difference spectra of cells by subtracting the mean spectrum of erlotinib-treated cells from that of control cells for each component identified by HCA. PCA was performed on the mean spectra for all subcellular components identified by HCA of both control and erlotinib-treated cells, and the first three PCs generated denote the higher variances within the data set [58]. PC1 loadings of respective subcellular components of control and erlotinib-treated cells were paired for comparison.

### Western blot

Phosphatase inhibitor/buffer mixture II (Sigma-Aldrich, Munich, Germany) and protease inhibitor cocktail (Roche) were used to harvest the cells. They were resolved by sodium dodecyl sulfate–10 % polyacrylamide gel electrophoresis, and transferred to Immobilon-P membranes (Millipore, Schwalbach am Taunus, Germany). The membranes were then incubated with antibodies to activate phosphorylated ERK1 and ERK2 (p-ERK; Cell Signaling Technology, Danvers, MA, USA), total ERK1 and ERK2 (Cell Signaling Technology, Danvers, MA, USA), phosphorylated AKT (p-AKT; Cell Signaling Technology, Danvers, USA), and total AKT (Cell Signaling Technology, Danvers, USA). Antibodies were detected with the appropriate anti-mouse horseradish peroxidase conjugated secondary antibody enhanced by chemiluminescence (Pierce, Life Technologies, Darmstadt, Germany), and images were captured with a Versa Doc 5000 imaging system (Bio-Rad, Munich, Germany).

## Results and discussion

### Downstream signalling and oncogenic mutations by Western blot

**Fig. 2:**
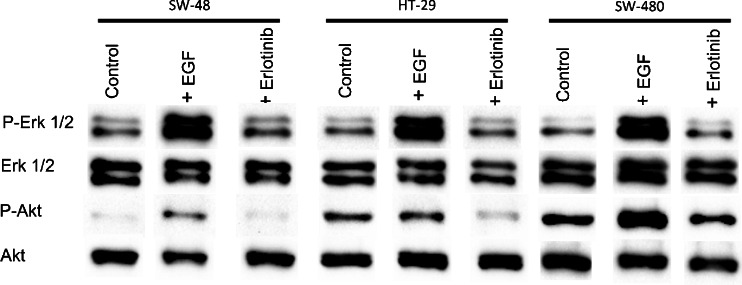
Effect of EGF (50 ng/ml for 15 min) and erlotinib (10 μg/ml for 2 h) on ERK and AKT activation in SW-48, HT-29, and SW-480 colon cancer cells. Cell lysates were resolved by sodium dodecyl sulfate–Polyacrylamide gel electrophoresis and Western blot analysis with use of antibodies that recognise phosphorylated ERK1 and ERK2 (*p-Erk 1/2*) and phosphorylated AKT (*p-Akt*) or total ERK1 and ERK2 (*Erk 1/2*), and AKT (*Akt*)

Binding of EGF to EGFR leads to dimerisation of EGFR, followed by phosphorylation of the EGFR TK domain. Subsequently, it leads to activation of the RAS machinery, which in turn activates multiple pathways towards cell proliferation (Fig. 1) [59,60]. Erlotinib blocks the receptor phosphorylation and consequently prevents RAS activation and inhibits cancer cell proliferation [10,11]. We performed Western blot experiments to detect the effect of erlotinib on the phosphorylation of two RAS downstream signalling pathways, RAF–ERK and PI3K–AKT, as shown in Fig. 2. It is expected that *BRAF* mutation in HT-29 cells and *KRAS* mutation in SW-480 cells can confer activation of p-ERK and p-AKT in control cells [61]. This activation is shown in Fig. 2. Although SW-48 cells harbour wild type *KRAS* and *BRAF*, they also show activation of p-ERK. This is because SW-48 cells carry a mutant *EGFR* allele [62]. EGF binding to EGFR increases the ERK and AKT phosphorylation level in these cancer cells.

Furthermore, no significant difference in p-ERK level was observed for erlotinib-treated SW-48, HT-29 and SW-480 cells compared with the control. This is perhaps due to the activating mutations of *BRAF* in HT-29 cells and *KRAS* in SW-480 cells as well as the *EGFR* mutation in SW-48 cells. Similar results were observed for the p-AKT level for erlotinib-treated SW-48 and SW-480 cells. On the other hand, the p-AKT level was reduced in erlotinib-treated HT-29 cells. This means that the Western blotting results did not significantly discriminate between the *KRAS* and *BRAF* status in these cells. These results are also in agreement with a study conducted to detect the effect of erlotinib on p-ERK and p-AKT in a series of pancreatic cancer cell lines with wild-type and mutated *KRAS* [63].

### Effect of erlotinib on SW-48 cells by Raman spectral imaging

Erlotinib is a reversible competitive inhibitor of the TK domain of EGFR that binds to its adenosine 5′-triphosphate binding site. To detect the effect of erlotinib binding to the TK domain, several Raman measurements of SW-48 cells were performed. Cells were incubated with erlotinib at 10 μg/ml for 12 h. The concentration of erlotinib used in this study is similar to that detected in the plasma of cancer patients taking this drug. The oral dosage of erlotinib in clinics is 150 mg/day, and the erlotinib concentration in the plasma was found to be 2.5–29 μg/ml [64]. No contribution from erlotinib to the Raman spectra itself was expected because of its low concentration, which cannot be detected under our experimental conditions.

**Fig. 3:**
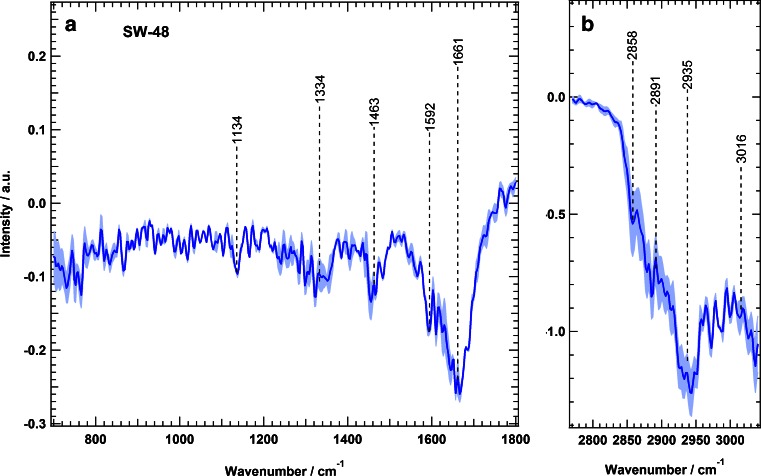
Raman difference spectra of SW-48 cells for control cells and erlotinib-treated cells in two spectral regions: *a* 700–1800 cm^−1^ and *b* 2800–3100 cm^−1^. The *shading* represents the standard deviation

We calculated the Raman difference spectrum of SW-48 cells by subtracting the mean spectrum of erlotinib-treated cells from that of the control cells; the results are presented in Fig. 3. The difference spectrum shows large spectral changes, which are represented by negative peaks at 1134 cm^−1^ (C–C/C–N stretching), 1334 cm^−1^ (amide III), 1463 cm^−1^ (C–H and CH_2_ bending deformation), 1592 cm^−1^ (C = C bending), 1661 cm^−1^ (amide I), and 2858, 2891, and 2935 cm^−1^ (C–H stretching) [42,65,66]. These changes can be attributed to the changes in proteins, nucleic acids, lipids, and carbohydrates that are constituents of the cell resulting from erlotinib treatment. Erlotinib-driven biochemical changes of subcellular components would result in cell cycle arrest and eventually lead to apoptosis [6,67–69].

**Fig. 4:**
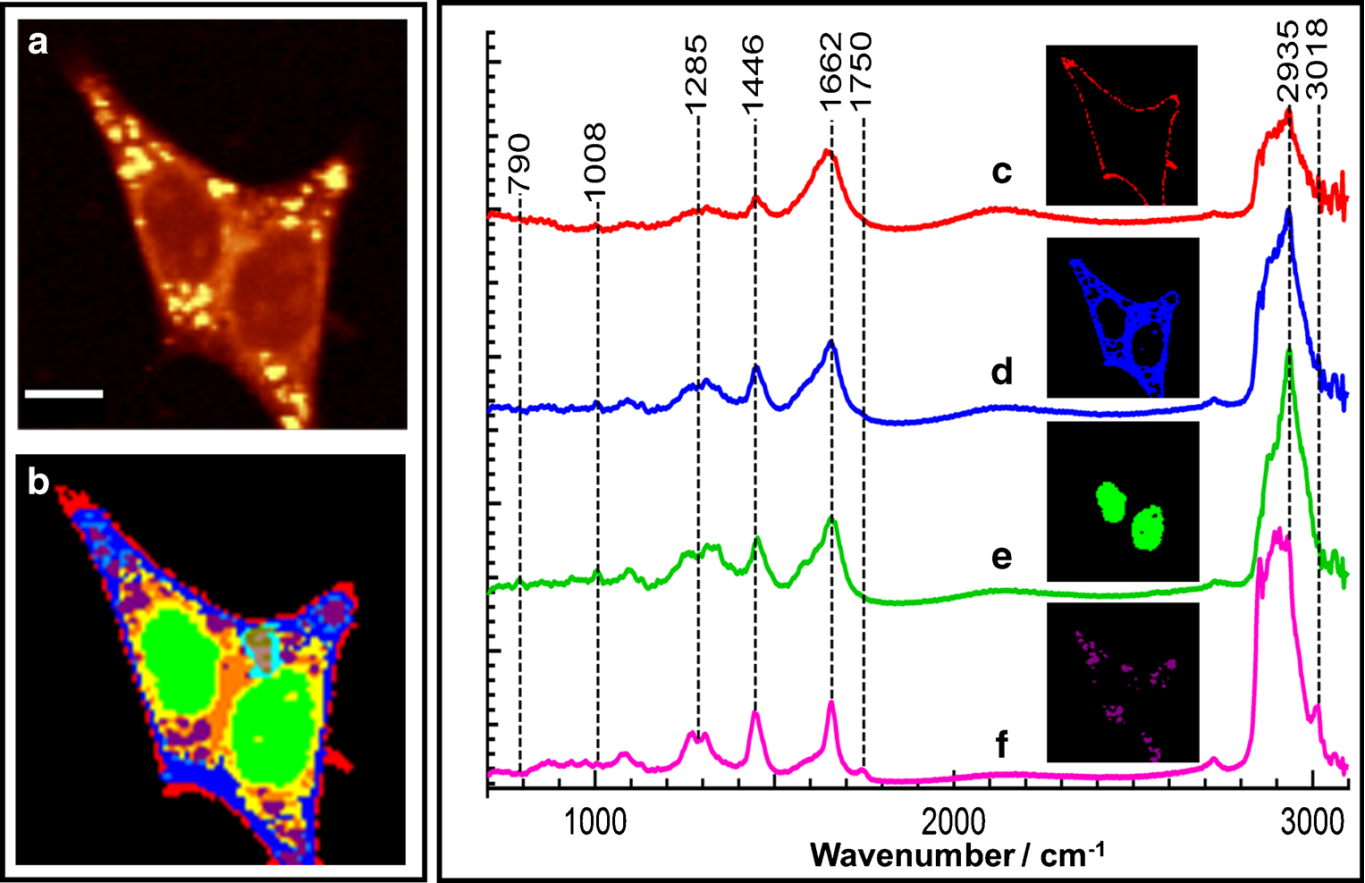
*a* Raman intensity image constructed in the C–H stretching region of SW-48 cells. b hierarchical cluster analysis (HCA) results based on the Raman data shown in *a*. Mean spectra and the respective spatial distribution of HCA clusters: *c* plasma membrane, *d* cytoplasm, *e* nucleus, and *f* lipid droplets. The *scale bar* in *a* is 9 μm

These results encouraged us to improve the spatial resolution of the cell response to erlotinib. We intended to emphasise the changes of subcellular components in response to erlotinib treatment. Subcellular organelles were determined by Raman spectroscopy coupled with HCA. Figure 4 shows a Raman intensity image of SW-48 cells in the absence of erlotinib (control) based on the integrated Raman intensities of the C–H stretching vibration in the 2800–3100-cm^−1^ region. The size of the image is 45 × 45 μm^2^ (90 × 90 pixels).

Next, HCA was performed in the spectral ranges from 700 to 1800 cm^−1^ and 2800 to 3100 cm^−1^ in order to construct a cluster map based on the Raman intensity image (Fig. 4, panel a); the result is shown in Fig. 4, panel b. The clusters were then merged to four major subcellular components: plasma membrane, cytoplasm, nucleus, and lipid droplets. This merging process was based on use of the spectral marker bands of each cell component. Afterwards, mean spectra of the four clusters were calculated (Fig. 4, spectra c–f). The outermost cluster (Fig. 4, spectrum c) is considered to contain the plasma membrane. However, the plasma membrane is smaller than the laser spot (less than 1 μm); therefore, the membrane cluster might contain contributions from the buffer and cytoplasm (Fig. 4, spectrum d). The mean spectrum of the nucleus (Fig. 4, spectrum e) contains characteristic Raman bands such as the stretching vibration of DNA phosphodiester at 790 cm^−1^. The spectrum of the cytoplasm (Fig. 4, spectrum d) and that of the nucleus (Fig. 4, spectrum e) both contain the Raman spectral band of the ring-breathing mode of phenylalanine at 1008 cm^−1^, which represents their protein constituent. However, cytoplasm spectra do not contain the DNA marker band at 790 cm^−1^ [55–57,70]. The Raman mean spectrum of lipid droplets (Fig. 4, spectrum f) shows characteristic bands of the C = O stretching vibration of the ester form of fatty acids at 1750 cm^−1^ and the = C―H stretching vibration of unsaturated fatty acids at 3018 cm^−1^. The spectra of the nucleus and lipid droplets are consistent with those reported previously [42,43,49,50].

**Fig. 5:**
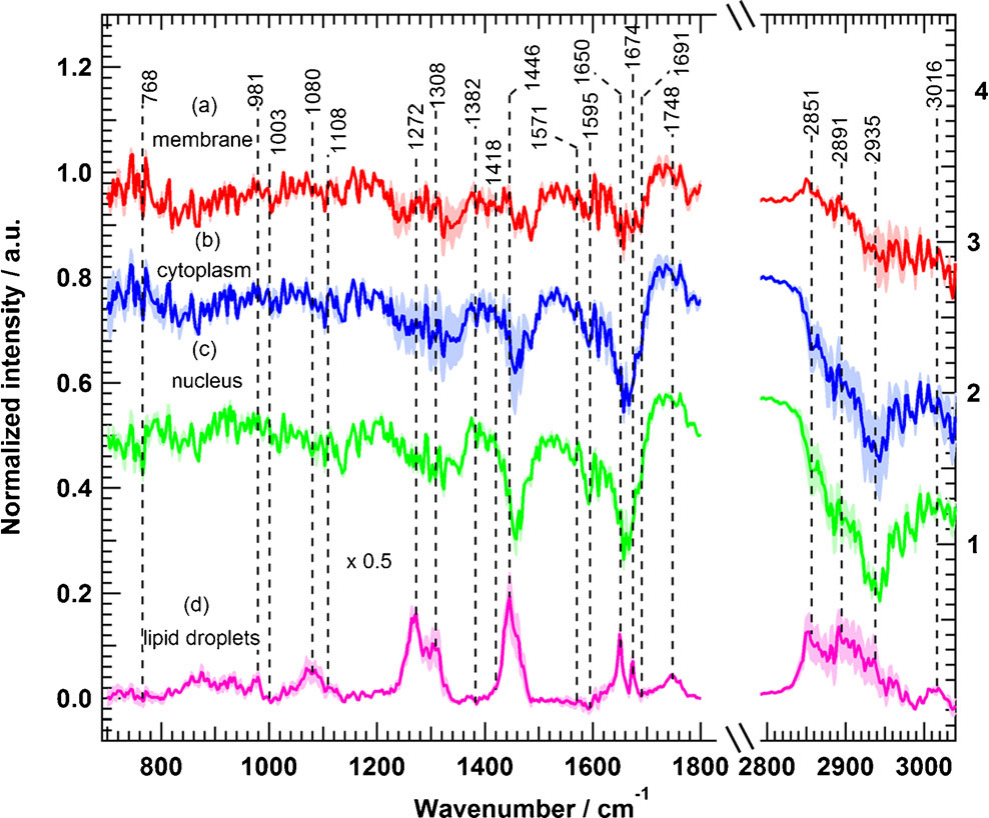
Raman difference spectra of SW-48 cells for control cells and erlotinib-treated cells for *a* plasma membrane, *b* cytoplasm, *c* nucleus, and *d* lipid droplets. The *shading* represents the standard deviation

To monitor the effect of erlotinib on cells, several Raman measurements of control cells (SW-48) and cells treated with erlotinib were acquired. Data analysis and HCA were performed for all Raman measurements in ways similar to those for the data presented in Fig. 4. All Raman mean spectra obtained from HCA were normalised with the phenylalanine band near 1008 cm^−1^. We calculated Raman difference spectra of cellular components by subtracting the mean spectrum of erlotinib-treated cells from that of control cells for each component identified by HCA; the results are shown in Fig. 5. Obvious spectral changes of the four cellular components were detected. In addition, the pairwise loading spectra from PCA of each component displayed clear differences on erlotinib treatment (see Figs. S1–S4). For example, the Raman difference spectrum of the membrane (Fig. 5, spectrum a) indicates negative peaks at 1231–1259 cm^−1^ (amide III, and PO_2_^−^, phospholipid region), 1321 cm^−1^ (CH_2_ twisting), 1458–1483 cm^−1^ (CH deformation), 1642–1688 cm^−1^ (amide I), and 2851 and 2891 cm^−1^ (C–H stretching). These spectral differences refer to changes in the lipid and protein constituents of the plasma membrane. The cytoplasm difference spectrum (Fig. 5, spectrum b) displays larger changes than that of the membrane, and shows negative peaks at 1231–1259 cm^−1^ (amide III), 1321 cm^−1^ (CH_2_ twisting), 1455 cm^−1^ (C–H deformation), 1592 cm^−1^ (C = C bending), 1642–1688 cm^−1^ (amide I), and 2851–2950 cm^−1^ (C–H stretching). In the case of the nucleus, the Raman difference spectrum (Fig. 5, spectrum c) reveals clear changes at 1134 cm^−1^ (C–C/C–N stretching), 1231–1259 cm^−1^ (amide III), 1321 cm^−1^ (CH_2_ twisting), 1458 cm^−1^ (C–H deformation), 1592 cm^−1^ (C = C bending), 1657–1666 cm^−1^ (amide I), and 2851–2950 cm^−1^ (C–H stretching).

**Fig. 6:**
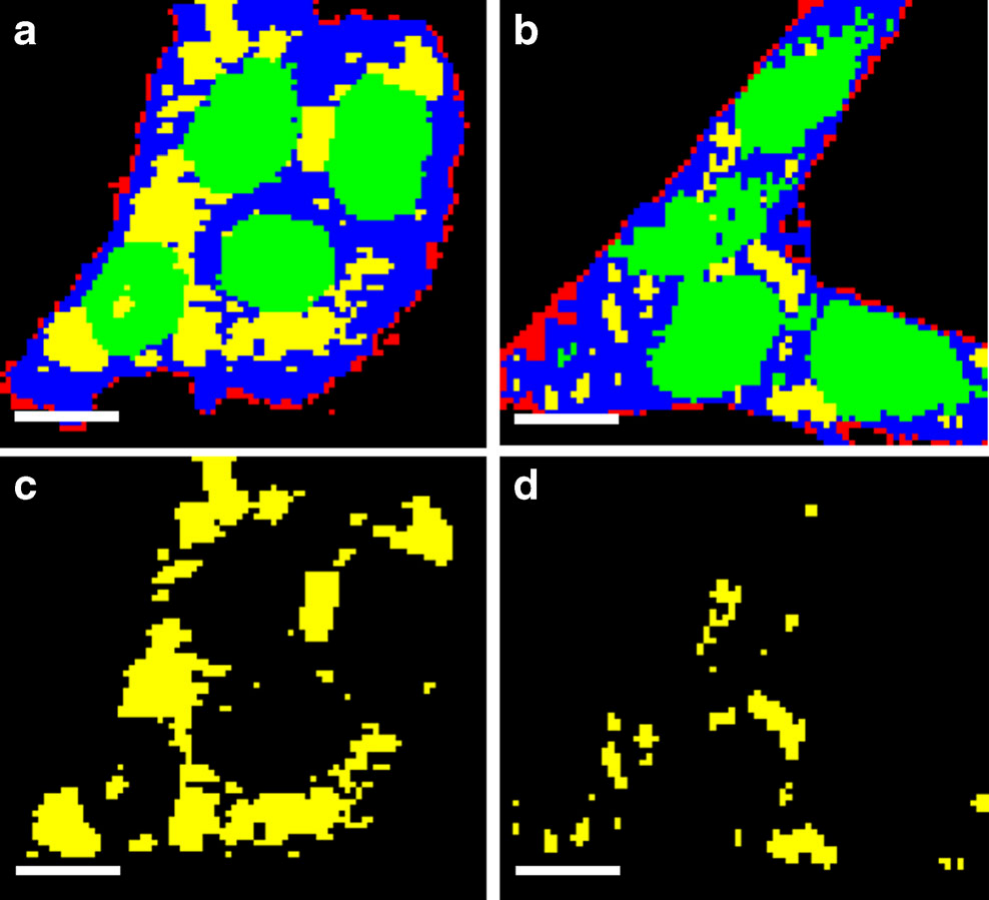
HCA results based on the Raman intensity images of **a** SW-48 control cells and **b** SW-48 erlotinib-treated cells. The clusters are shown in *red* for plasma membrane, *blue* for cytoplasm, green for nuclei, and *yellow* for lipid droplets. **c** HCA cluster of lipid droplets from **a. d** HCA cluster of lipid droplets from **b**. The *scale bar* is 7 μm

The largest changes of all the cell components were observed in the Raman difference spectrum of lipid droplets (Fig. 5, spectrum d). It exhibits intense positive peaks at 1272 cm^−1^ (CH_3_ twisting), 1308 cm^−1^ (CH_2_ twisting), 1446 cm^−1^ (C–H deformation), 1650–1674 cm^−1^ (C = C– stretching vibration of unsaturated fatty acids), 1744 cm^−1^ (=C–H stretching vibration of unsaturated fatty acids), 2851–2950 cm^−1^ (CH stretching), and 3016 cm^−1^ (=C–H stretching). These strong positive peaks refer to a decrease in the intensity of the mean spectrum of lipid droplets in erlotinib-treated cells. This might be produced as a result of a decrease in the number of lipid droplets in erlotinib-treated cells. In fact, lipid droplets play a major role in cell proliferation. It was believed that the only function of lipid droplets is to store and metabolise lipids. However, it is found that lipid droplets are also involved in protein storage, degradation, and trafficking processes [71]. Lipid droplets are also accumulated in cancer cells, and they induce proliferation in colon cancer [72,73]. In addition, lipid droplets decrease in number or disappear in cells treated with drugs, and the amount of lipid droplets might have some effect in drug resistance of tumour cells [74]. Even more, erlotinib treatment results in reducing accumulation of lipid droplets in human cardiomyocytes [75]. Thus, lipid droplets have become an important target in cancer therapy [72,73]. HCA images based on Raman measurements of SW-48 cells show that the number of lipid droplets decreases in cells treated with erlotinib (Fig. 6b, d) in comparison with control cells (Fig. 6a, c). Given these facts, we suggest that the positive peaks of lipid droplets in the difference spectrum (Fig. 5d) are produced as a result of reduction of lipid droplet numbers in erlotinib-treated cells when compared with control cells. In contrast to lipid droplets, the Raman difference spectra revealed negative peaks for the membrane, cytoplasm, and nucleus on erlotinib treatment, implying that the Raman intensity of these components is increased in erlotinib-treated cells. This is perhaps due to an increase in the expression level of proteins during apoptosis on erlotinib treatment [68,69,76].

### Resistance of oncogenic mutations to erlotinib therapy by Raman spectral imaging

**Fig. 7:**
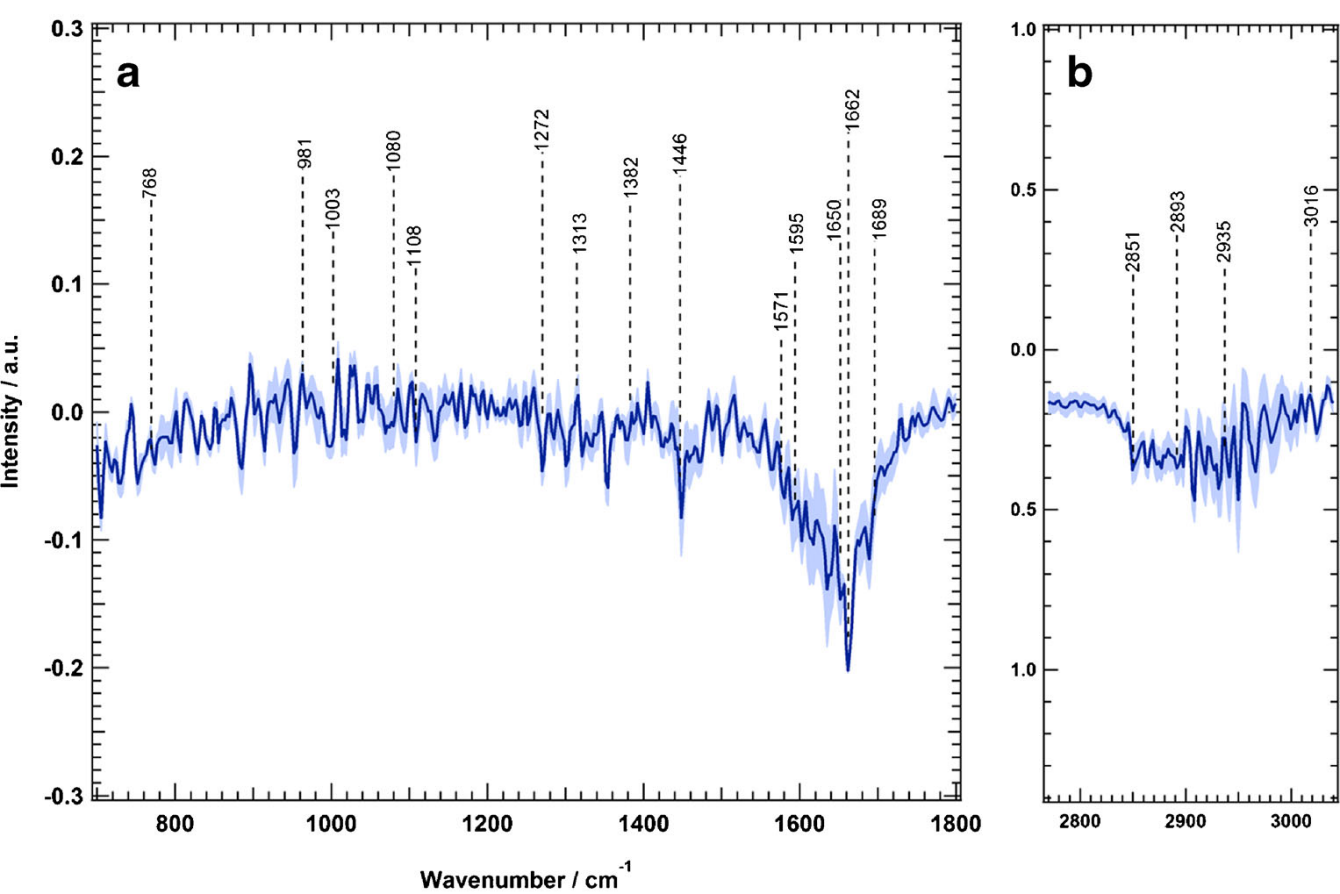
Raman difference spectra of HT-29 cells for control cells and erlotinib-treated cells in two spectral regions: *a* 700–1800 cm^−1^ and *b* 2800–3100 cm^−1^ . The *shading* represents the standard deviation

**Fig. 8:**
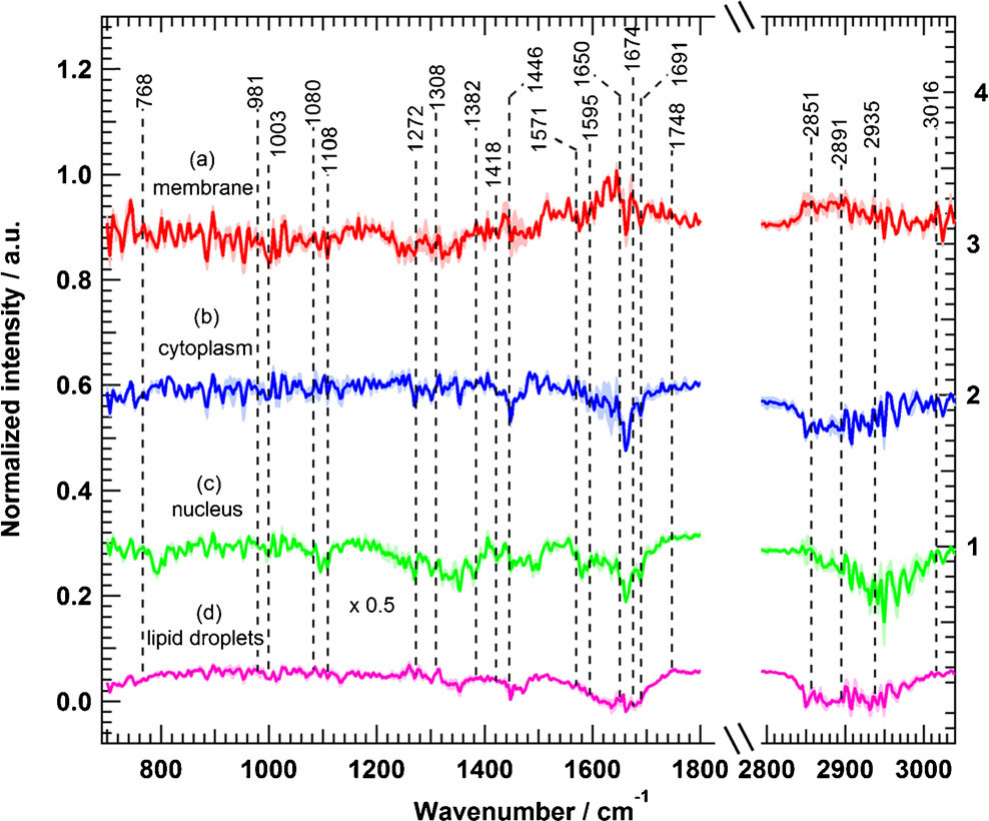
Raman difference spectra of HT-29 cells for control cells and erlotinib-treated cells for *a* plasma membrane, *b* cytoplasm, *c* nucleus, and *d* lipid droplets. The *shading* represents the standard deviation

We applied the experimental conditions used for SW-48 cells to the other two colon cancer cell lines, HT-29, and SW-480, which have oncogenic mutations of *BRAF* (V600E), and *KRAS* (G12V), respectively [77]. Figure 7 displays the Raman difference spectrum of HT-29 cells, which shows relatively few spectral differences when compared with the Raman difference spectrum of SW-48 cells (Fig. 3). Spectral changes are observed (Fig. 7) at 1446, 1595–1690, 2851, 2893, and 2935 cm^−1^. Further clustering of four subcellular components of HT-29 cells was performed, and the Raman difference spectra detect some small spectral changes in the plasma membrane, cytoplasm, nucleus, and lipid droplets, as indicated in Fig. 8.

**Fig. 9:**
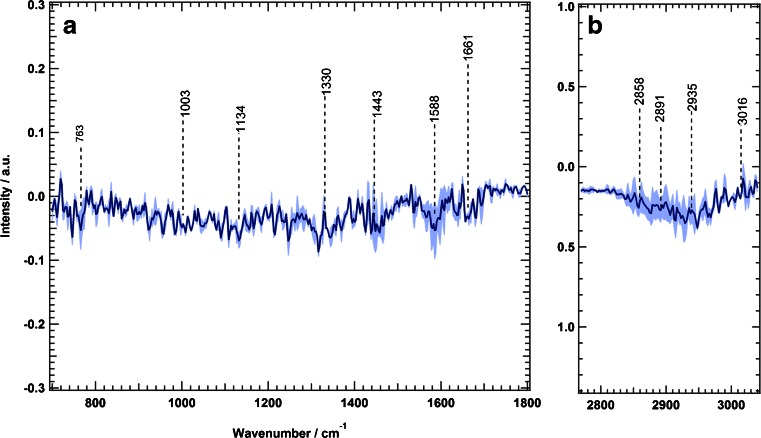
Raman difference spectra of SW-480 cells for control cells and erlotinib-treated cells in two spectral regions: *a* 700–1800 cm^−1^ and *b* 2800–3100 cm^−1^. The *shading* represents the standard deviation

In the case of the membrane (Fig. 7, spectrum a), the Raman difference spectrum shows changes at 1446, 1600–1660, and 2851–3935 cm^−1^. The cytoplasm difference spectrum (Fig. 7, spectrum b) displays negative spectral changes at 1448, 1650–1674, and 2851–2935 cm^−1^. In addition, the nucleus difference spectrum (Fig. 7, spectrum c) shows negative spectral changes at 1095, 1300–1360, 1583, 1650–1690, and 2891–2980 cm^−1^. Finally, the lipid droplet spectrum (Fig. 7, spectrum d) exhibits notable changes at 1272, 1595–1690, 1748, and 2851–2935 cm^−1^. To compare the observed spectral changes for both SW-48 cells (Fig. 3 or Fig. 5) and HT-29 cells (Fig. 7 or Fig. 8), the same intensity scale is used. HT-29 cells (Figs. 7 and 8), harbouring *BRAF* mutation, showed a small response to erlotinib treatment in comparison with SW-48 cells (Figs. 3, 5). Furthermore, Fig. 9 shows the calculated Raman difference spectrum of SW-480 cells. The spectrum reveals no significant spectral changes between control and erlotinib-treated cells. Thus, the Raman results show that HT-29 cells (*BRAF* mutated) and SW-480 cells (*KRAS* mutated) display a smaller and not significant change, respectively, on erlotinib treatment.

It is worth mentioning that *BRAF* mutation affects mainly one signalling pathway for cell proliferation, whereas *KRAS* mutation affects multiple pathways (Fig. 1); therefore, the impact of *BRAF* mutation is less than that of *KRAS* mutation. In agreement with the present Raman results, clinical studies on human patients harbouring *BRAF*- or *KRAS*-mutated tumours have shown a limited response to TK inhibitors [22,25,29,34,78]. On the other hand, the Western blot results could not discriminate between erlotinib-treated cells harbouring wild-type *KRAS* and *BRAF* and those with *KRAS* and *BRAF* mutations through monitoring the ERK phosphorylation. In addition, the AKT phosphorylation is inhibited only in cells with *BRAF* mutations. In Western blot, antibodies are used to mark single proteins although cellular proteins are highly coupled in protein networks, and monitoring of a single protein may not reflect the in vivo cellular response to drugs such as erlotinib. This might explain the differences between the findings of in vitro drug studies and the drug effect observed in cancer patients. On the other hand, Raman microscopy reflects the overall chemical composition of the cell, and Raman difference spectra can detect the changes in the whole cell contents, which would be an efficient way to exhibit the overall cellular and subcellular component changes upon drug treatment.

Developing a new preclinical method that would predict the resistance of mutated cancers to therapy can reduce the cost, time, and effort spent in the new drug discovery process. It is one step further to increase the accuracy of in vitro drug evaluation, before these drugs proceed to clinical trials to be tested on human patients. In fact, clinical trials suffer from many ethical and economical limitations [5]. Consequently, increasing the accuracy of in vitro evaluation tests would have a significant impact on accelerating the process of new anticancer drug evaluation and final approval.

## Conclusion

Raman microscopy coupled with clustering analysis such as HCA can be used to detect the subcellular changes induced by drug treatment. Raman difference spectra can also emphasise the oncogenic mutational drug resistance in EGFR-targeted therapy. Erlotinib resistance in *BRAF*- and *KRAS*-mutated colon cancers is clearly reflected in their Raman difference spectra. Cancer cells with wild-type *KRAS* and *BRAF* show clear changes upon erlotinib treatment, whereas *BRAF*- and *KRAS*-mutated cancer cells display a limited change or no significant changes, respectively. Consistently, clinical studies revealed that human cancer patients with *BRAF*- or *KRAS*-mutated tumours have the worst therapy prognosis. Accordingly, in vitro Raman imaging is a valuable addition that can enhance the sensitivity of the preclinical evaluation methods for new anticancer agents.

## Electronic supplementary material

ESM 1 (PDF 1.86 mb)
