# Supplementary material for: In vitro prediction of the efficacy of molecularly targeted cancer therapy by Raman spectral imaging
Source: Anal Bioanal Chem. 2015 Jul 14;407(27):8321–31. doi: 10.1007/s00216-015-8875-z (PMC4604500; doi:10.1007/s00216-015-8875-z)
Supplement: Supplementary file 1 — (PDF 1.86 mb) [file 216_2015_8875_MOESM1_ESM.pdf]

Analytical and Bioanalytical Chemistry

Electronic Supplementary Material

In vitro prediction of the efficacy of molecularly targeted cancer therapy by Raman spectral imaging

Hesham K. Yosef, Laven Mavarani, Abdelouahid Maghnouj, Stephan Hahn,
Samir F. El-Mashtoly, Klaus Gerwert

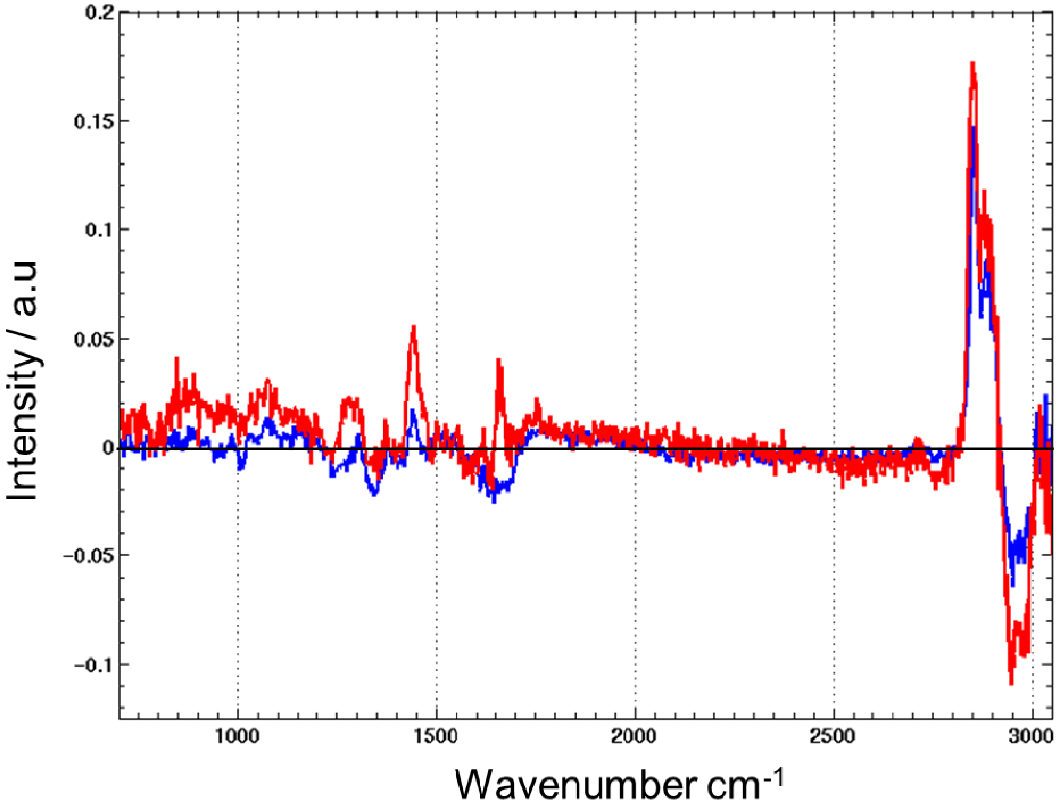

Fig. S1 Plot of paired loadings resulting from the principle component analysis of the membrane mean spectra of both control cells (red) and erlotinib-treated cells (blue)

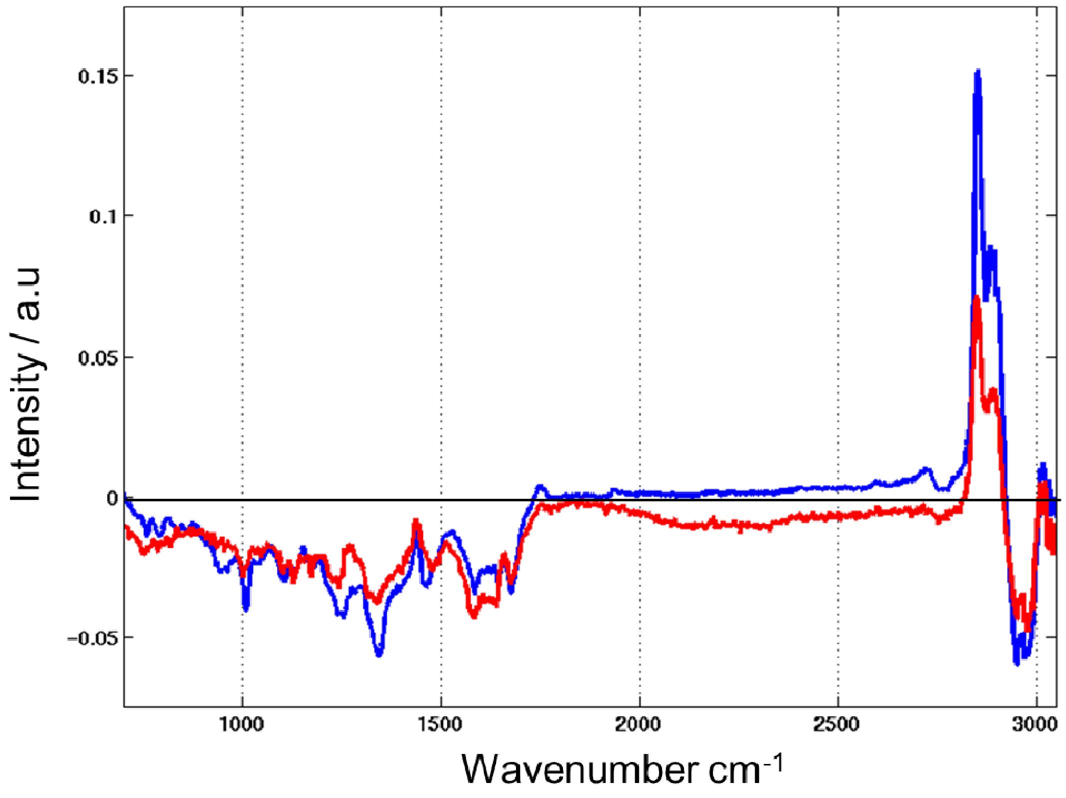

Fig. S2 Plot of paired loadings resulting from the principle component analysis of the cytoplasm mean spectra of both control cells (red) and erlotinib-treated cells (blue)

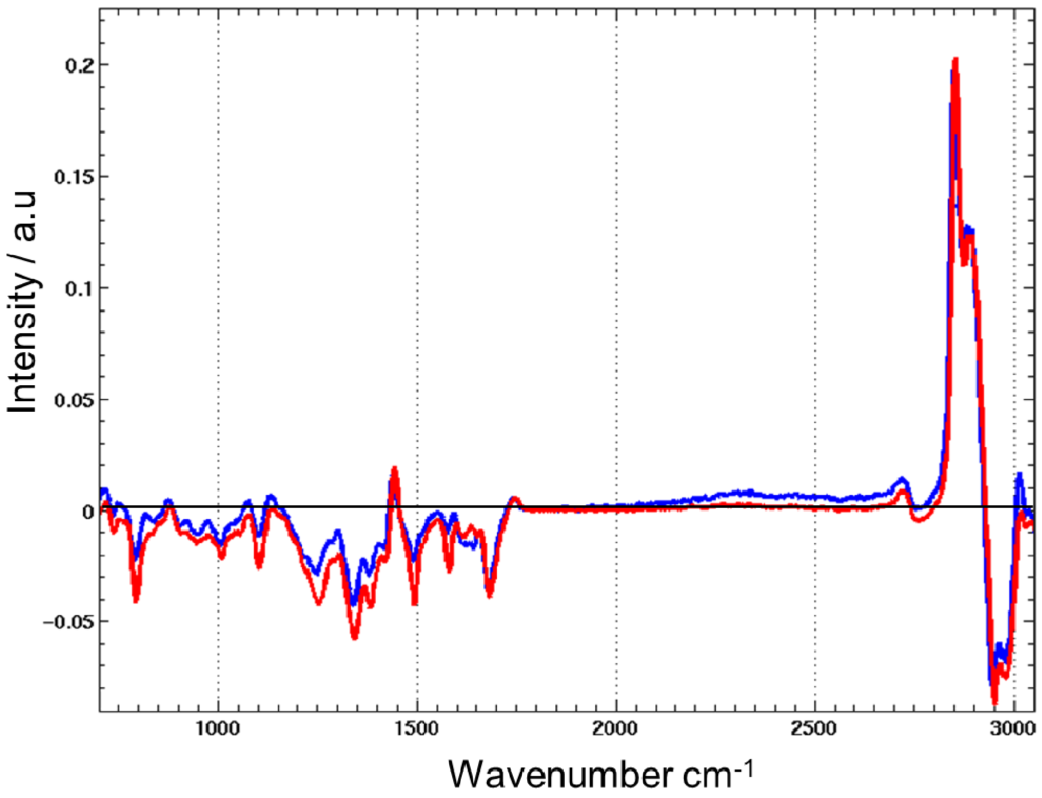

Fig. S3 Plot of paired loadings resulting from the principle component analysis of the nucleus mean spectra of both control cells (red) and erlotinib-treated cells (blue)

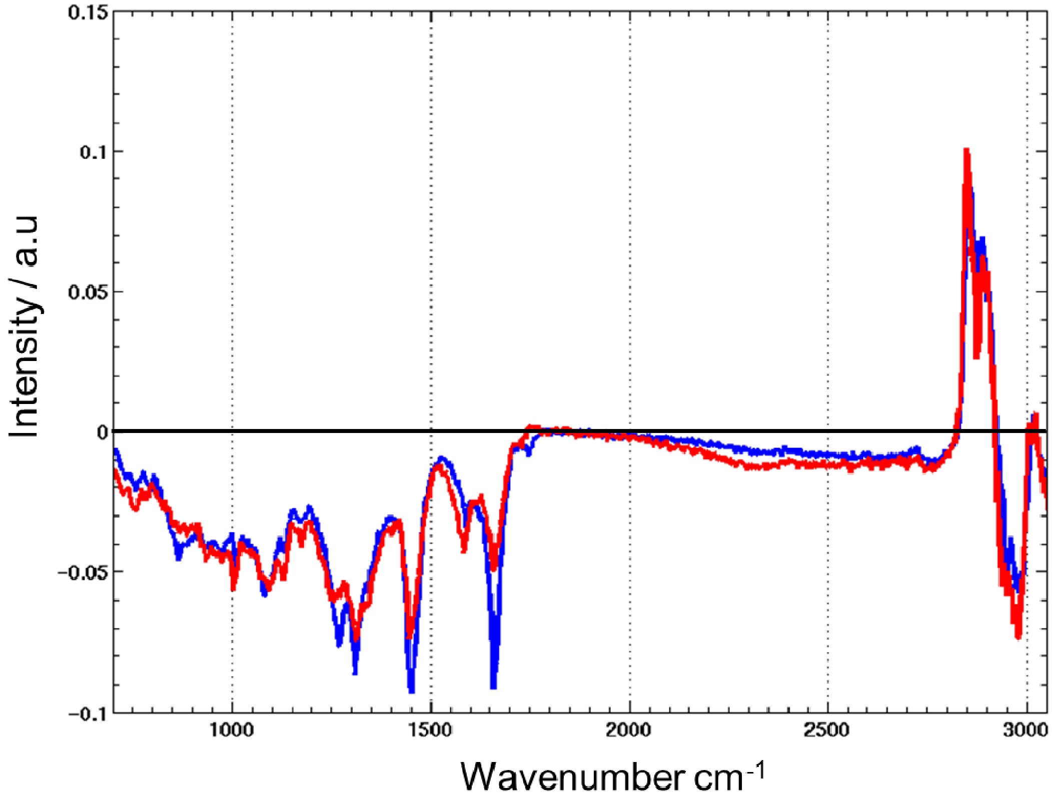

Fig. S4 Plot of paired loadings resulting from the principle component analysis of the lipid droplets mean spectra of both control cells (red) and erlotinib-treated cells (blue)
